# Isolation and genomic analysis of phage BUCT551 against drug-resistant *Aeromonas hydrophila*

**DOI:** 10.3389/fvets.2025.1679093

**Published:** 2025-09-26

**Authors:** Pengjun Han, Hongbo Qin, Chenxi Hao, Zhiping Du, Yang Yang

**Affiliations:** ^1^Department of Neurosurgery, Zhumadian Central Hospital, Affiliated Central Hospital of Huanghuai University, Zhumadian, China; ^2^College of Medicine, Huanghuai University, Zhumadian, China; ^3^College of Life Science and Technology, Beijing University of Chemical Technology, Beijing, China

**Keywords:** *Aeromonas hydrophila*, *Aeromonas veronii*, drug resistant, phage BUCT551, genome analysis

## Abstract

*Aeromonas hydrophila* (*A. hydrophila*) is a common pathogen in aquaculture that also causes opportunistic infections and sporadic food- and water-borne illness in humans. Phage therapy is increasingly considered a promising complementary medicine for antibiotic therapy. In this study, we isolated a novel *A. hydrophila* phage (designated BUCT551) using *A. hydrophila* strain Ah18 as an indicator. The one-step growth curve demonstrated that BUCT551 had a latent period of 20 min and a burst size of 32 PFU/cell at its optimal multiplicity of infection (0.1). BUCT551 had a survival pH range from 5 to 10 and could tolerant temperatures from 4 °C to 50 °C. Host range analysis showed that the phage was able to lyse not only *A. hydrophila*, but also *Aeromonas veronii*. Whole-genome sequencing of BUCT551 revealed a linear DNA genome of 61,382 bp. Bioinformatics analysis demonstrated that the genome of phage BUCT551 contains 74 predicted open reading frames (ORFs), of which 27 were annotated as functional proteins with assigned biological roles. Notably, no lysogeny-associated genes, antimicrobial resistance determinants, virulence factors, or tRNA genes were identified in this phage genome. A comparative genomic analysis showed that phage BUCT551 is the closest relative to *Aeromonas* phage LAh_7 and shares the same new branch in the phylogenetic tree. Characterization of the phage BUCT551 enriches our knowledge about the diversity of *A. hydrophila* phages.

## 1 Introduction

*Aeromonas hydrophila* (*A. hydrophila*) is a Gram-negative, non-spore-forming, rod-shaped, facultative anaerobic organism belonging to the *Aeromonadaceae* family (1). It is ubiquitous in the natural environment because of its wide range of survival and reproductive temperatures (from 4 °C to 40 °C) (2). *A. hydrophila* is usually found in aquatic environments, such as fresh river water, and is recognized as an opportunistic pathogenic of poikilothermic and homoeothermic animals including humans (3, 4). *A. hydrophila* is considered the causative agent of multiple diseases in many fish species, including ulcerative disease, hemorrhagic disease, red sore disease, and septicemia, leading to severe economic losses to the aquaculture industry (5–7). Furthermore, *A. hydrophila* is pathogenic to humans, especially immunocompromised individuals, as it colonizes the human intestinal tract and has been linked to gastroenteritis, skin diseases, and septicemia (5, 8–10). *A. hydrophila* produces virulence factors, including aerolysin, hemolysin, adhesion factor, enterotoxin, and extracellular protease, that act individually or in synergy, resulting in tissue damage and contributing to pathogenicity (8). *Aeromonas* infections have been reported in recent decades, and data from Japan indicated that *Aeromonas* species were isolated from 5% of travelers (23,215 persons in total) who returned from developing countries (10). In 2018–2019, Hilt et al. isolated several multidrug-resistant *A. hydrophila* isolates from two solid organ transplant patients (11). In another recent case, Hasan et al. reported *A. hydrophila* infection in an immunocompromised 69-year-old woman, with surgical site sepsis/infection and several comorbidities (12).

The increasing levels of antibiotic resistance among bacteria present a great challenge to conventional medicine and pose a real threat to human life (13, 14). Antibiotic resistance among *Aeromonas* spp. has attracted increasing attention in recent years. Elbehiry et al. isolated *A. hydrophila* from 150 meat and water samples to screen their antibiotic resistance, and found that some *A. hydrophila* strains were resistant to ampicillin, cefotaxime, pefloxacin, ceftazidime, and ciprofloxacin (15). In another study, *A. hydrophila* was found to have acquired a gene encoding the carbapenemase GES-24, conferring carbapenem resistance (16). Given that *A. hydrophila* exhibits pathogenicity in both humans and animals, and outbreaks lead to economic losses in agriculture/aquaculture or public health incidents, the emergence of antibiotic resistance in this pathogen should be a focus of research.

As a result of increasing antibiotic drug resistance, phage therapy has gained attention, with the potential for use either alone or in combination with antibiotics (17–19). Phages are exclusively virulent against prokaryotic microbes and do not lyse animal and human cells. They can be classified into lytic phage and temperate phage depending on their life cycle. Lytic phages lyse host bacteria immediately after undergoing replication and assembly in the host, leading to the elimination of host bacteria. The characteristics of lytic phages endow therapeutic potential against bacterial infection (20). Some phages demonstrate high specificity to a single species or even a certain strain of bacteria (21). To enhance the efficacy when using phages as antibacterial agents, a strategy has been suggested that involves mixing a cocktail of different phages into one formulation (22). However, only a few *A. hydrophila* phages have been isolated and characterized (23). In this study, we isolated and characterized a novel phage specific to drug-resistant *A. hydrophila*. Additionally, we evaluated the application potential of this phage by characterizing its physiological properties, lytic activity, host range, and genomic features. This systematic assessment provides a comprehensive basis for determining its suitability in therapeutic scenarios.

## 2 Materials and methods

### 2.1 Bacterial strain isolation and identification

The host strain Ah18 was isolated from an aquaculture farm (Nanjing, China). Molecular identification of strain Ah18 was conducted based on 16S rRNA gene sequencing. The gene was amplified from genomic DNA using the universal primers 27F (5′-AGAGTTTGATCCTGGCTCAG-3′) and 1492R (5′-GGCTACCTTGTTACGACTT-3′). The resulting amplicons were sequenced by RuiBiotech (Beijing, China). The obtained sequence was subjected to BLASTn analysis via the NCBI database, which revealed 100% identity with the 16S rRNA sequence of *A. hydrophila* strain GSH8-2 (accession no. AP019193.1). Based on these results, we confirmed that Ah18 belonged to species *A. hydrophila*. Strain Ah18 was cultured at 28–30 °C with shaking at 220 × *g* in Tryptic Soy Broth (TSB) medium (BD Difco).

### 2.2 Drug sensitivity test of *A. hydrophila* strain Ah18

The antimicrobial susceptibility profile of strain Ah18 was determined using the broth microdilution method as previously described (24). The antibiotics used in the test were purchased from Fosun Diagnostics (Shanghai, China). The bacterial isolate Ah18 was cultured on Mueller–Hinton (MH) agar and incubated at 28 °C for 24 h. A bacterial suspension adjusted to 0.5 McFarland standard was prepared in sterile saline, diluted with MH broth to approximately 1 × 10^6^ CFU/mL, and 50 μL of the diluted suspension was aliquoted into each well of a microplate. Two wells were designated as controls: one containing only bacterial suspension (positive control) and another containing only sterile MH broth (negative control). The microplate was incubated at 28 °C for 24 h. *Escherichia coli* ATCC 25922 and *Staphylococcus aureus* ATCC 29213 were used as quality control strains and incubated at 37 °C. The minimum inhibitory concentration (MIC) was defined as the lowest antimicrobial concentration that completely inhibited visible bacterial growth. MIC values were interpreted as sensitive, intermediate, or resistant according to the Clinical and Laboratory Standards Institute (CLSI) guidelines (CLSI VET03/VET04-S2, 2014).

### 2.3 Phage isolation and purification

The sewage sample collected from Nanjing aquatic market was centrifuged at 12,000 × g for 10 min, then the supernatant was filtered through a 0.22-μm membrane. The phage filtrate was pipetted into a culture solution containing *A. hydrophila* strain Ah18 at logarithmic phase. The mixture was incubated overnight at 28 °C in a shaker to allow the phages to proliferate. The phage stock was obtained by centrifugation at 12,000 × g for 10 min at 4 °C. To verify the phage stock, a sample was applied onto double-layer agar plates, with the upper agar mixing with Ah18, and the plates were incubated until the appearance of plaques. After verification, purification of the phage isolate was performed as previously described (25). The diluent at optimum concentration was mixed with host bacteria to generate double-layer agar plates. After incubation, single plaques were selected and cultured in TSB medium using Ah18 as an indicator. Phages were re-harvested by centrifugation and filtration. The purification processes were conducted three times.

### 2.4 Transmission electron microscopy

Transmission electron microscopy (TEM) was performed to determine the morphological characteristics of phages after concentration using cesium chloride (CsCl) gradient centrifugation (26). A phage suspension was pipetted onto carbon-coated support membrane copper mesh, left for 20 min, then negatively stained with 2% phosphotungstic acid solution for a 1-min retention time. After further drying, phage particles were observed under an electron microscope (JEM-1200EX, Japan) at an accelerating voltage of 100 kV.

### 2.5 Determination of multiplicity of infection

The multiplicity of infection (MOI) signifies the ratio of the number of phage in a system to the total number of bacteria and it can be interpreted as the number of phage added to each cell during infection. A single colony of strain Ah18 was inoculated into 5 mL of fresh TSB medium and cultured with shaking at 28 °C until reaching the early-exponential phase (approximately 1 × 10^7^ CFU/mL). Subsequently, different concentrations of phage particles, diluted with sterile TSB, were separately introduced into parallel bacterial cultures to achieve MOI of 0.01, 0.1, 1, 10, 100. The mixtures were then incubated with shaking at 28 °C for 12 h. Finally, the phage titers under each MOI condition were determined using the double layer agar method (27).

### 2.6 Thermal stability and pH sensitivity

To investigate the vigor of phage BUCT551 under multiple environmental conditions, we measured the phage titers across diverse temperatures and pH values using previously described methods (25). In the thermal stability assay, phages (approximately 10^9^ PFU/mL) were incubated in a water bath at different temperatures (4 °C, 30 °C, 40 °C, 50 °C, 60 °C, 70 °C, 80 °C) for 1 h, then the phage titers were determined by the double layer agar method. For pH stability assessment, 100 μL of phage suspension (2 × 10^10^ PFU/mL) was mixed with 900 μL of TSB pre-adjusted to pH 3–12 using NaOH or HCl. Following incubation at 28 °C for 1 h, then the phage titers were calculated by the double layer agar method. All experiments were performed in triplicate and the results are presented as the mean value.

### 2.7 One-step growth curve assay

The one-step growth curve assay was performed to determine the eclipse period and burst size of phage BUCT551. Phage BUCT551 was mixed with *A. hydrophila* strain Ah18 at exponential phase (approximately 8 × 10^7^ CFU/mL) and optimal MOI, then 30 min absorption at 28 °C was performed. The mixture was centrifuged at 12,000 × *g* for 1 min to separate free phage particles within host bacteria immediately after the absorption procedure. The harvested cells were washed twice with sterile TSB and resuspended in 50 mL of TSB medium. Subsequently, the mixture was cultured with shaking at 28 °C for 205 min and samples were collected at 0, 10, 20, 30, 40, 55, 70, 85, 100, 115, 130, 145, 160, 175, 190, and 205 min during incubation. The phage titers were determined by the double-layer agar method as described above. According to the one-step growth curve, the latent and lysis periods for the phage were obtained. The burst size of phage BUCT551 was calculated using the following formula: Burst size (PFU/cell) = (Final phage titer at plateau phase) / (Initial number of infected host bacteria) (28).

### 2.8 Host range analysis

The bacterial susceptibility level was inspected using spotting method as previously described (26). Eight strains of *A. hydrophila* (Ah 154, ATCC 419140, AS1. 18031, Ah 7, Ah 9, Ah 10, Ah 17, Ah 18, No. 201308071) and eight strains of *A. veronii* (Av 4, Av 6, Av 15, Av 26, Av 25, Av 13, Av 21, Av 3) that were stored at the Biosafety Technology Research Center, Beijing University of Chemical Technology, were selected for host range determination. In brief, 500 μL of the *A. hydrophila* culture was transferred to 5 mL of TSB medium containing 0.75% agar, which was promptly poured onto a tryptic soy agar (TSA) plate. After solidification, 2.5 μL of phage BUCT551 was pipetted onto the double-layer agar, then the plates were cultured overnight to observe plaque formation. Additionally, the susceptibility of the tested bacterial strain to phage BUCT551 was determined using the efficiency of plaquing (EOP) assay (29). EOP values were calculated by normalizing plaque formation efficiency on the target strain relative to that observed on the reference strain Ah18.

### 2.9 Genome extraction, sequencing and analysis

The genome of phage BUCT551 was extracted using the proteinase K/SDS method as described by Yang et al. (30). Subsequently, the DNA library was constructed using NEBNext Ultra II FS DNA Library Prep Kit (NEB) as manufacturer’s instructions. The concentration of nucleic acid was determined using Qubit 2.0 (Invitrogen, United States). Whole-genome sequencing of phage BUCT551 was performed using the MiSeq platform (Illumina, United States). Newbler v3.13.0 software (Roche 454) and CLC software (CLC Bio) were used for genomic sequence assembly (31). Low-quality sequences were filtered out using the Trimmomatic (v0.32) program (32). The Phred score cut-off was set at Q ≥ 30, with a scanning window size of 20 bp, and a minimum read length cut-off of 50 bp.

Possible protein-coding genes were initially predicted using the Rapid Annotation using Subsystem Technology server.^1^ The predicted open reading frames (ORFs) were confirmed with the ORF Finder,^2^ and their putative functions were annotated using BLASTp.^3^ The genome function map was generated by the laboratory’s self-built script and modified using Inkscape 0.92.3.0. The genomic sequences of phage BUCT551 and phage LAh_7 were aligned using the Easyfig 2.2.3 (33). The tRNAs in the phage BUCT551 genome were predicted using the software tRNAscan-SE v.2.0 (34). The possible virulence and pathogen genes carried by phage genome were predicted with the VFDB (35) and PathogenFinder (36), respectively.

A phylogenetic tree was constructed based on the amino acid sequence of the conserved major capsid and terminase large subunit to analyze the relationship between phage BUCT551 and other phages of the same subfamily recorded in the International Committee on Taxonomy of Viruses database (ICTV). MEGA version 7.0 was employed to construct the phylogenetic tree (37).

### 2.10 *In vitro* antibacterial curve determination

The antibacterial activity of phage BUCT551 was assessed according to a previously established method (38). Briefly, exponential-phase *A. hydrophila* Ah18 cultures (1 × 10^8^ CFU/mL) were co-incubated with an equal volume of phage suspensions at different MOIs of 0.01, 0.1, 1, and 10, respectively. The mixtures were incubated with shaking at 28 °C for 12 h. Untreated *A. hydrophila* cultures (supplemented with an equivalent volume of TSB broth) served as controls. Bacterial growth was monitored by measuring optical density at 600 nm (OD₆₀₀) at each time point. All experiments were performed in triplicate.

### 2.11 Statistical analysis

GraphPad Prism 8.0.1 (GrapPad Software, San Diego, CA, United States) was used to plot the data. One-way analysis of variance (ANOVA) was used to evaluate the difference between the experimental and control groups. The *p*-value < 0.05 was considered statistically significant.

## 3 Results

### 3.1 Drug sensitivity analysis of *A. hydrophila* Ah18

Antimicrobial susceptibility testing of *A. hydrophila* isolate Ah18 was conducted using antibiotics commonly employed in aquaculture. The results revealed distinct resistance in Ah18, specifically to florfenicol, thiamphenicol, and doxycycline (Table 1). This finding underscores that prolonged antibiotic usage in aquaculture can drive bacterial resistance development, ultimately limiting the efficacy of antimicrobial therapies.

**Table 1:** Antimicrobial susceptibility of *A. hydrophila* isolate Ah18.

Drug types	Drug name	MIC (μg/mL)	Susceptibility
Sulfonamides	Sulfamonomethoxine	128	S
	Trimethoprim/sulfamethoxazole	2/38	S
4-quinolones	Flumequine	1	S
	Enrofloxacin	0.25	S
Chloramphenicols	Florfenicol	512	R
	Thiamphenicol	512	R
Tetracyclines	Doxycycline	32	R
Aminoglycosides	Neomycin	0.5	S

S, sensitivity; R, resistance.

### 3.2 Phage morphology

**Figure 1:**
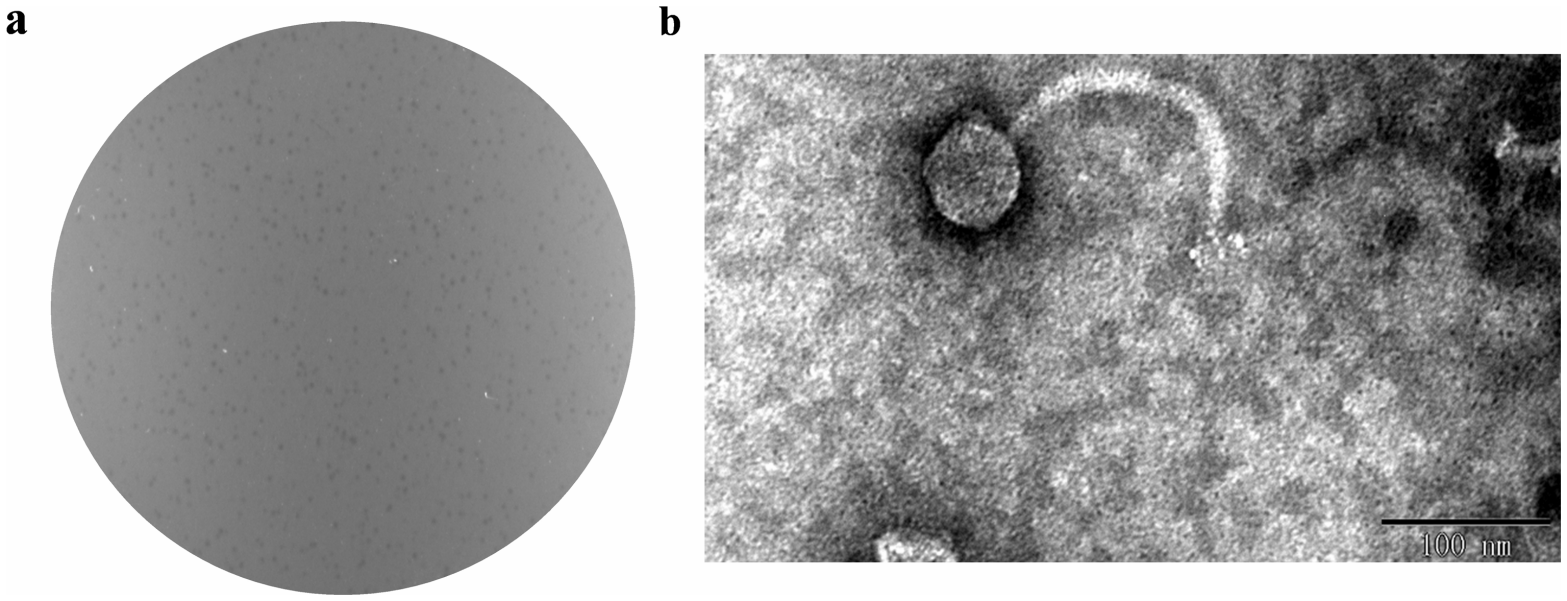
The morphology of phage BUCT55. **(a)** Plaques formed by phage BUCT551 on *A. hydrophila* strain Ah18 using the double agar overlay method. **(b)** Transmission electron micrograph of phage BUCT551. Scale bar: 100 nm.

An *A. hydrophila* phage was isolated from aquacultural sewage and was designated BUCT551. Phage BUCT551 formed clear plaques of 0.5–1 mm in diameter after 8 h incubation on the lawn of *A. hydrophila* strain Ah18 (Figure 1a). TEM revealed that phage BUCT551 had a polyhedral capsid (50 ± 3 nm in diameter) and a long tail (180 ± 5 nm) (Figure 1b).

### 3.3 Thermal stability and pH sensitivity

**Figure 2:**
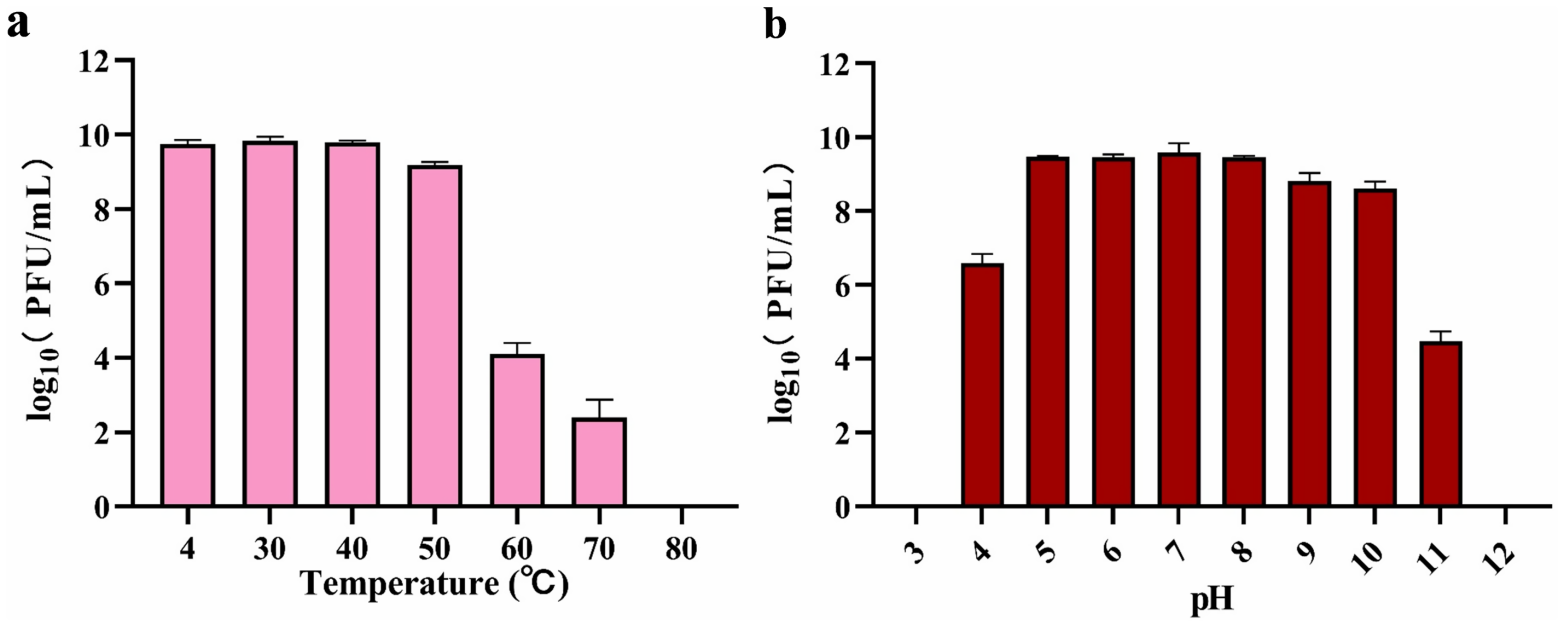
Stabilities of phage BUCT551 under **(a)** temperature 4 °C to 80 °C and **(b)** pH 3 to 12. Results are presented as mean values ± SD.

A thermal stability assay confirmed that phage BUCT551 could survive across a temperature range of 4–50 °C. The survival rate decreased significantly over 50 °C, and the phage was inactivated completely after incubating at 80 °C for 1 h (Figure 2a). The pH sensitivity assay confirmed that phage BUCT551 exhibited relative stability within the pH range of 5–10. Phage titers decreased dramatically after 1 h of incubation at pH < 5 or pH > 10, with complete inactivation observed at pH 3 and pH 12 (Figure 2b). Phage activity was significantly reduced following exposure to pH 3 and pH 12, indicating loss of infectivity under these extreme conditions.

### 3.4 Optimal MOI and one-step growth curve

**Figure 3:**
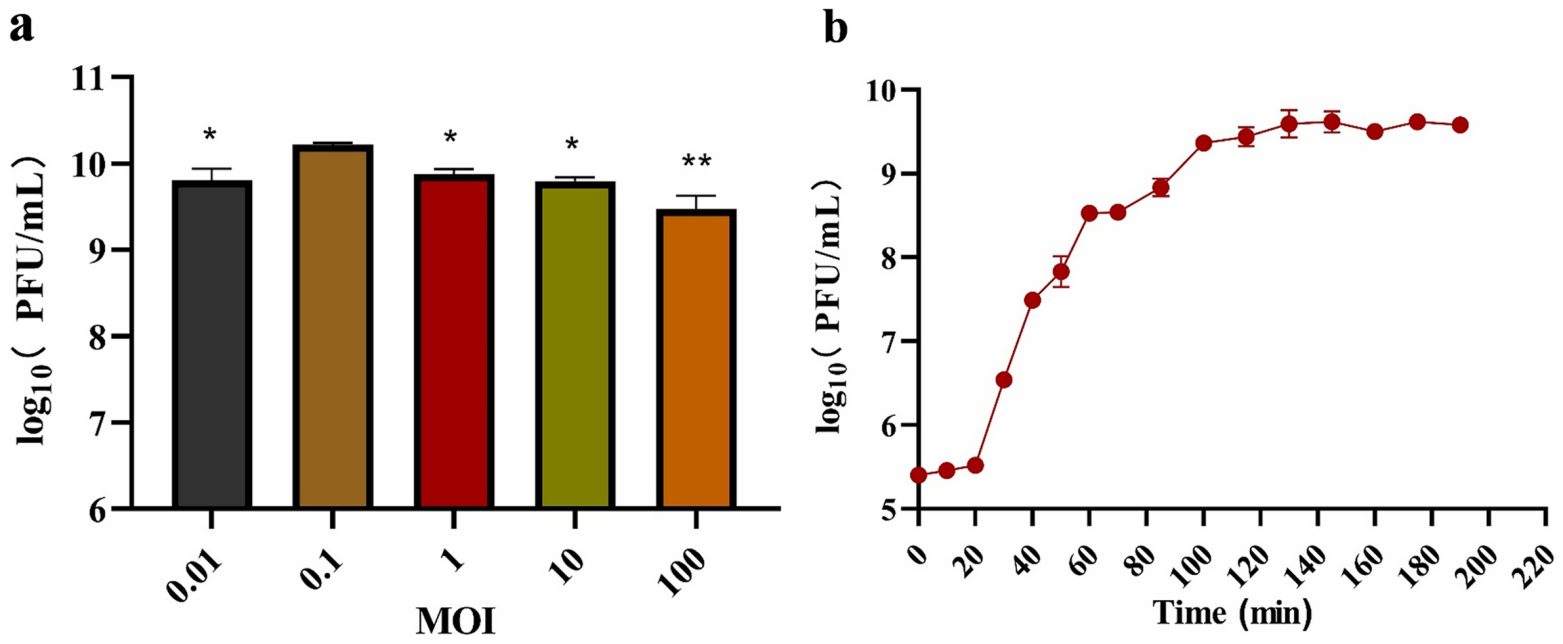
Growth characterization of phage BUCT551. The determination results of the **(a)** optimal MOI and **(b)** one-step growth curve. Results are presented as mean values ± SD, *p < 0.05 or **p < 0.01 indicates a significant difference compared to the MOI 0.1.

The titer of phage BUCT551 was tested under different MOIs infection conditions. On the basis of the maximum titer obtained after phage BUCT551 infection of *A. hydrophila* strain Ah18, the optimal MOI of the phage was 0.1 (Figure 3a). A one-step growth curve assay revealed that phage BUCT551 had a latent period of 20 min and a growth period of 80 min, and reached a plateau at 100 min, when the phage titer was 2.6 × 10^9^ PFU/mL (Figure 3b). According to the formula described in the Methods section, the burst size of phage BUCT551 was calculated to be 32 PFU/cell.

### 3.5 Host range analysis

In order to understand the lysis range of phage BUCT551, all eight strains of *A. hydrophila* and eight strains of *A. veronii* in our laboratory were selected for examination. The results indicated that one strain of *A. hydrophila* and four strains of *A. veronii* were susceptible to phage BUCT551, suggesting that BUCT551 has therapeutic potential for *Aeromonas* infection (Table 2). Unfortunately, phage BUCT551 did not exhibit lytic activity against *Vibrio*, a genus closely related to *Aeromonas*, which reflects its narrow host specificity (Supplementary Table S1).

**Table 2:** Host range analysis of the phage BUCT551.

Strains	Genus, species	Susceptibility	EOP
Av 3	*Aeromonas, veronii*	+	74
Av 4	*Aeromonas, veronii*	−	0
Av 6	*Aeromonas, veronii*	−	0
Av 13	*Aeromonas, veronii*	−	0
Av 15	*Aeromonas, veronii*	−	0
Av 21	*Aeromonas, veronii*	+	21
Av 25	*Aeromonas, veronii*	+	0.45
Av 26	*Aeromonas, veronii*	+	1.6
Ah 7	*Aeromonas, hydrophila*	−	0
Ah 9	*Aeromonas, hydrophila*	−	0
Ah 10	*Aeromonas, hydrophila*	−	0
Ah 17	*Aeromonas, hydrophila*	−	0
Ah 18	*Aeromonas, hydrophila*	+	100
No.2013080717	*Aeromonas, hydrophila*	−	0
ATCC 419140	*Aeromonas, hydrophila*	−	0
AS1.18031,	*Aeromonas, hydrophila*	−	0

“+” denotes the formation of transparent plaques following co-culture of phage BUCT551 with the test bacterium; “−” indicates no plaque formation. EOP is calculated in percent as the PFU/mL of the phages on the test strain divided by the PFU/mL obtained on strain Ah18 multiplied by 100.

### 3.6 Whole-genome analysis

**Figure 4:**
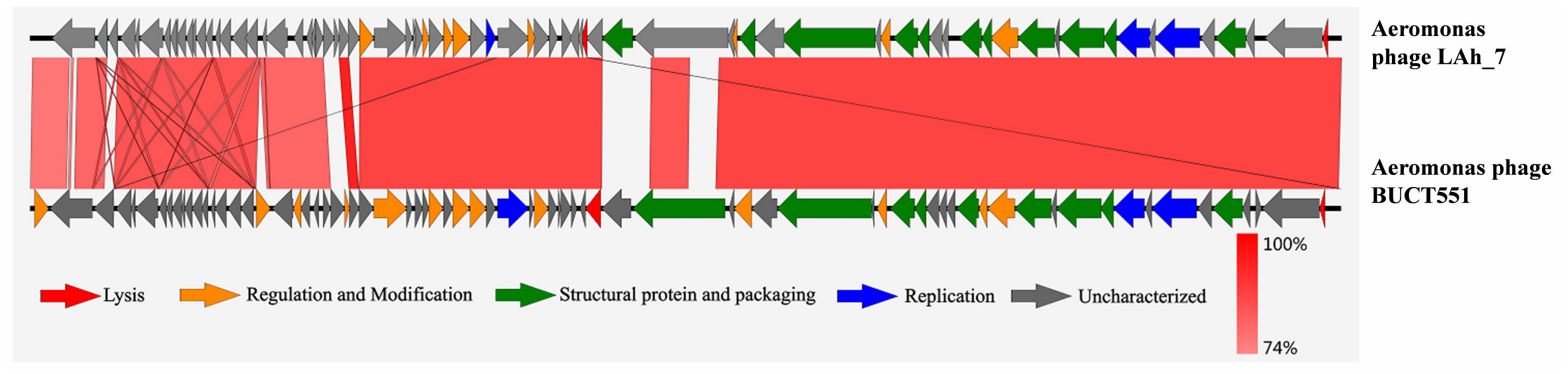
The homology comparison between phage BUCT551 and phage LAh_7. The gradual red vertical bar in the lower right corner of the figure indicates the genetic homology between BUCT551 and LAh_7 in the picture.

Whole-genome sequencing revealed that BUCT551 has a linear genome of 61,382 bp with a GC content of 61.77%. Restriction endonucleases specifically act on double-stranded DNA, and therefore restriction endonuclease digestion followed by electrophoresis can be employed to characterize nucleic acids. The enzyme-digested phage BUCT551 genome exhibited multiple electrophoretic bands, which preliminarily verified that BUCT551 is a double-stranded DNA phage (Supplementary Figure S1). BLASTn analysis revealed that phage BUCT551 shares 86.75% homology (87% genome coverage) with *A. hydrophila* phage LAh_7. Comparative analysis of the genomic sequences between bacteriophages BUCT551 and LAh_7 was conducted using deduced amino acid sequences of all ORFs. The results revealed significant similarity at the protein level (Figure 4). Notably, sequence identity in the 3′-terminal region of the genome, which encodes multiple proteins with unknown functions, was relatively lower than the genome-wide average. This observed divergence may represent adaptive mutations acquired by the bacteriophages in response to evolutionary pressures within their respective ecological niches (39).

### 3.7 Functional open reading frame analysis

RAST gene annotation identified 74 ORFs within the phage BUCT551 genome. ATG was the initiation codon for most ORFs. Among the 74 predicted protein coding genes, 27 were identified as functional coding sequences, with the remainders annotated as proteins of unknown function or hypothetical proteins (Table 3). Further bioinformatic analysis revealed that the bacteriophage BUCT551 genome harbors no lysogeny-associated genes, antimicrobial resistance determinants, virulence factors, or tRNA genes. These findings collectively indicate that BUCT551 demonstrates genomic safety at the nucleotide level, fulfilling a critical prerequisite for its potential therapeutic application.

**Table 3:** Predicted ORFs in the genome of phage BUCT551.

ORFs	Strand	Start	Stop	Best-match BLASTp result	Query cover	*E*-values	Gen Bank Acc. No
ORF1	+	210	866	HNH endonuclease	100%	4e-112	QFG04404.1
ORF2	−	2,902	920	Hypothetical protein	76%	0.0	QDH46647.1
ORF3	−	3,915	2,986	Hypothetical protein	99%	3e-166	QFG04406.1
ORF4	−	4,721	4,044	Hypothetical protein LAh7_4	100%	3e-142	QDH46667.1
ORF5	−	4,921	4,721	Hypothetical protein LAh7_5	100%	2e-35	QDH46713.1
ORF6	−	5,979	4,918	Hypothetical protein LAh7_6	100%	7e-172	QDH46657.1
ORF7	−	6,379	6,047	Hypothetical protein	98%	6e-63	QFG04410.1
ORF8	−	6,656	6,381	Hypothetical protein	100%	5e-43	QFG04411.1
ORF9	−	7,096	6,653	Hypothetical protein LAh7_9	98%	1e-75	QDH46683.1
ORF10	−	7,249	7,100	Hypothetical protein	100%	8e-08	QFG04413.1
ORF11	−	7,584	7,249	Hypothetical protein LAh7_11	100%	2e-66	QDH46694.1
ORF12	−	8,051	7,587	Hypothetical protein LAh7_12	100%	9e-88	QDH46680.1
ORF13	−	8,329	8,048	Hypothetical protein LAh7_13	83%	2e-25	QDH46701.1
ORF14	−	8,663	8,475	Hypothetical protein LAh7_14	93%	7e-09	QDH46710.1
ORF15	−	9,189	8,656	Hypothetical protein LAh7_15	100%	2e-95	QDH46675.1
ORF16	−	9,859	9,266	Hypothetical protein	100%	6e-129	QFG04419.1
ORF17	−	10,466	9,870	Hypothetical protein LAh7_17	100%	1e-114	QDH46672.1
ORF18	+	10,591	11,244	DNRLRE domain-containing protein	81%	5e-25	TPV95153.1
ORF19	−	12,302	11,334	Hypothetical protein LAh7_19	100%	2e-135	QDH46659.1
ORF20	−	12,687	12,313	GntR family transcriptional regulator	100%	2e-28	QFG04422.1
ORF21	−	13,093	12,677	Hypothetical protein LAh7_20	100%	6e-79	QDH46687.1
ORF22	−	13,516	13,265	Hypothetical protein LAh7_21	100%	9e-54	QDH46706.1
ORF23	+	13,713	14,054	Hypothetical protein LAh7_23	96%	1e-54	QDH46695.1
ORF24	+	14,074	14,730	Hypothetical protein	100%	6e-122	QFG04426.1
ORF25	+	14,727	14,942	RES domain-containing protein	100%	4e-37	QFG04427.1
ORF26	+	14,942	15,403	Hypothetical protein LAh7_26	100%	2e-89	QDH46679.1
ORF27	+	15,400	16,074	Hypothetical protein LAh7_28	99%	4e-133	QDH46668.1
ORF28	+	16,085	17,647	Exonuclease	100%	0.0	QFG04431.1
ORF29	+	17,631	17,879	Hypothetical protein LAh7_30	95%	2e-49	QDH46699.1
ORF30	+	18,022	18,378	Hypothetical protein LAh7_32	100%	3e-68	QDH46691.1
ORF31	+	18,375	18,671	Hypothetical protein	100%	2e-18	QFG04434.1
ORF32	+	18,668	19,348	Putative 3′-5′ exoribonuclease	100%	4e-142	QDH46666.1
ORF33	+	19,360	19,782	Hypothetical protein LAh7_35	100%	9e-96	QDH46684.1
ORF34	+	19,784	20,581	Putative 3′-5′ exoribonuclease	100%	2e-180	QDH46660.1
ORF35	+	20,595	21,356	Putative N-6-adenine-methyltransferase	98%	5e-173	QDH46661.1
ORF36	+	21,356	21,793	Hypothetical protein LAh7_38	100%	1e-73	QDH46686.1
ORF37	+	21,894	23,342	Putative DNAligase/BRCA1domainprotein	100%	0.0	QDH46651.1
ORF38	+	23,372	23,566	Hypothetical protein	81%	2e-09	QFG04441.1
ORF39	+	23,610	24,257	Putative 3′-phosphatase, 5′-polynucleotide kinase	100%	1e-137	QDH46665.1
ORF40	+	24,267	24,659	Hypothetical protein LAh7_42	99%	1e-81	QDH46690.1
ORF41	+	24,727	24,852	NO hit	100%	0.0	QAY02159.1
ORF42	+	24,855	25,346	Hypothetical protein LAh7_43	100%	1e-109	QDH46678.1
ORF43	+	25,343	25,594	Hypothetical protein	100%	1e-36	QFG04446.1
ORF44	−	26,010	25,762	Hypothetical protein LAh7_46	100%	4e-29	QDH46703.1
ORF45	−	26,714	26,007	Putative lysis protein A	97%	2e-155	QDH46662.1
ORF46	−	28,129	26,768	Hypothetical protein	46%	0.40	QFG04449.1
ORF47	−	32,540	28,224	Putative tail protein	63%	0.0	QDH46642.1
ORF48	−	32,952	32,728	Hypothetical protein LAh7_51	100%	6e-44	QDH46711.1
ORF49	−	33,783	32,962	DUF2163 domain-containing protein	100%	5e-138	QFG04452.1
ORF50	−	34,973	33,780	Hypothetical protein LAh7_53	100%	0.0	QDH46654.1
ORF51	−	39,412	34,973	Putative tape measure protein	97%	0.0	QDH46643.1
ORF52	−	39,518	39,405	Hypothetical protein	80%	1e-08	QFG04455.1
ORF53	−	40,111	39,662	Putative tail assembly chaperone	99%	1e-77	QDH46682.1
ORF54	−	41,414	40,269	Putative major tail protein	100%	0.0	QDH46656.1
ORF55	−	41,953	41,417	Putative minor tail protein	99%	2e-102	QDH46673.1
ORF56	−	42,557	41,946	Hypothetical protein LAh7_59	99%	4e-139	QDH46670.1
ORF57	−	42,904	42,554	Hypothetical protein	100%	5e-60	QFG04460.1
ORF58	−	43,315	42,908	Hypothetical protein	100%	2e-35	QFG04461.1
ORF59	−	44,412	43,372	Putative major capsid protein	100%	0.0	QDH46658.1
ORF60	−	44,817	44,428	Decorator protein	99%	1e-75	QFG04463.1
ORF61	−	46,103	44,820	Putative S49 family peptidase	100%	0.0	QDH46655.1
ORF62	−	47,815	46,100	Putative portal protein	100%	0.0	QDH46648.1
ORF63	−	48,054	47,815	Hypothetical protein LAh7_65	100%	1e-50	QDH46708.1
ORF64	−	50,153	48,051	Putative terminase large subunit	100%	0.0	QDH46646.1
ORF65	−	50,718	50,119	Putative terminase small subunit	99%	4e-135	QDH46671.1
ORF66	−	52,175	50,727	Putative ATP-dependent helicase	99%	0.0	QDH46649.1
ORF67	−	52,512	52,222	Hypothetical protein LAh7_69	100%	6e-60	QDH46696.1
ORF68	−	54,622	52,514	Putative DNA polymerase	100%	0.0	QDH46645.1
ORF69	−	55,312	54,683	Hypothetical protein LAh7_71	100%	9e-141	QDH46669.1
ORF70	−	56,772	55,363	DUF2800 domain-containing protein	100%	0.0	QFG04472.1
ORF71	−	57,128	56,772	Hypothetical protein LAh7_73	81%	8e-48	QDH46688.1
ORF72	+	57,385	57,654	Hypothetical protein	94%	1e-39	QFG04474.1
ORF73	−	60,365	57,684	Hypothetical protein	100%	0.0	QFG04475.1
ORF74	−	60,626	60,384	Putative AlpA family regulatory protein	100%	2e-48	QDH46707.1

**Figure 5:**
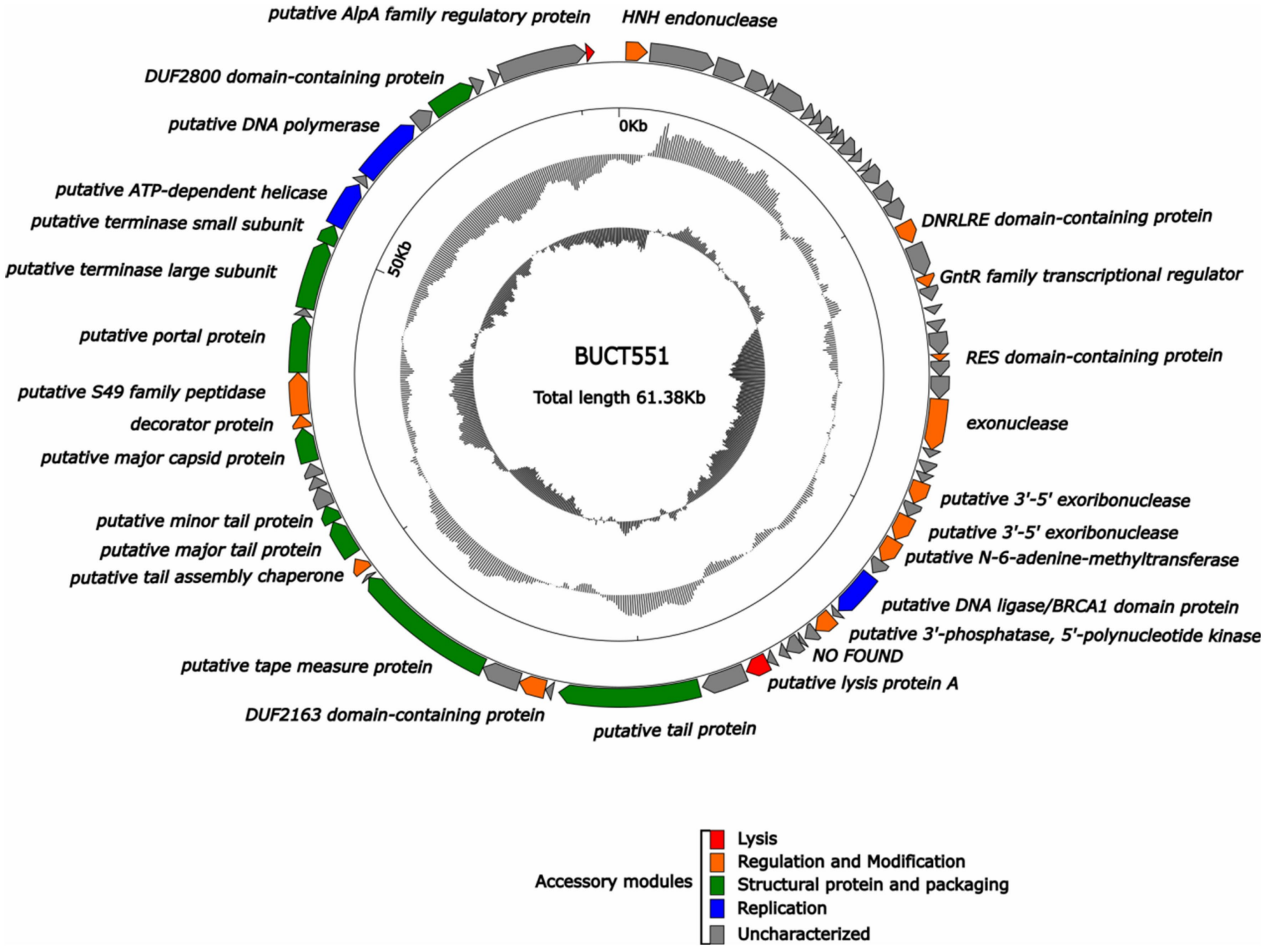
Whole genome map of BUCT551. The outermost layer displayed 74 open reading frames encoded in the genome, different colors represent different functions. The innermost circle represents the GC skew (G-C/G + C. Outwards indicates > 0 and inwards indicates < 0).

We classified the 27 proteins with annotated functions into four functional divisions: replication, modification and regulation, structural and packaging, and lytic division. The detailed annotation and organization of the BUCT551 genome is illustrated in Figure 5. Both BLASTp and RAST analyses demonstrated that ORF64 and ORF65 encode the large and small subunits of the terminase, respectively, which plays a significant role in phage DNA packaging. With regard to the phage replication division, ORF68 (DNA polymerase), ORF32 (putative 3′-5′ exoribonuclease), ORF34 (putative 3′-5′ exoribonuclease), and ORF39 (putative 3′-phosphatase, 5′-polynucleotide kinase), encoded by phage BUCT551, participate in the regulation of phage DNA replication. Furthermore, the DNA helicase encoded by ORF66 and the HNH endonuclease encoded by ORF1 have been speculated to play a crucial role in DNA replication and regulation, and subsequent modification (40). ORF35 encodes N-6-adenine-methyltransferase, a regulatory enzyme that is likely to participate in phage DNA modification, whose aim was to circumvent the protection system of host bacteria (41).

Phage structural proteins are encoded by multiple ORFs including ORF47 (putative tail protein), ORF51 (putative tape measure protein), ORF54 (putative major tail protein), ORF55 (putative minor tail protein), ORF53 (putative tail assembly chaperone), ORF59 (putative major capsid protein), and ORF62 (putative portal protein). Portal protein, encoded by ORF62, is considered to function in channel formation of the capsid and phage genome injection into host cells (42). The tape measure protein encoded by ORF51 plays a role in the assembly of phage capsid protein (43).

ORF18 (DNRLRE domain-containing protein), ORF20 (GntR family transcriptional regulator), ORF25 (RES domain-containing protein), ORF49 (DUF2163 domain-containing protein), ORF53 (putative tail assembly chaperone), ORF60 (decorator protein), and ORF61 (putative S49 family peptidase) encode proteins that play roles in phage modification and regulation. ORF53, encoding the tail assembly chaperone, exists widely in the genomes of *Siphoviridae* and *Myoviridae* phages, and is generally located between genes encoding the tail and tape measure proteins, thereby participating in regulation via frameshift mutations (44). ORF60 encodes a decoration protein. Decoration proteins are a class of proteins present in DNA viruses that locate on the surface of certain phage capsids, especially phages with double-stranded DNA genomes, where they protect the phage from extreme environmental changes by enhancing and promoting the robustness of the capsid (45). Many decoration proteins also possess other functions, such as facilitating target cell recognition, participating in phage assembly, and regulating host gene expression (45). Previous research has demonstrated that the GntR family of transcription factors encoded by ORF20 generally exist in prokaryotes and certain viruses and regulate various important metabolic pathways (46). The GntR family of transcription factors are also recognized to play a vital role in the growth and reproduction in bacteria (47).

ORF45 (putative lysis protein A) and ORF74 (putative AlpA family regulatory protein) belonging to the phage lytic division, probably encode lytic peptidases and enable the phage to lyse host cells effectively (48).

Notably, the majority of ORFs (47 in total) were classified as proteins of unknown function. Future research is warranted to clarify the function of these proteins to gain further insight into this phage.

### 3.8 Phylogenetic tree analysis for phage BUCT551

**Figure 6:**
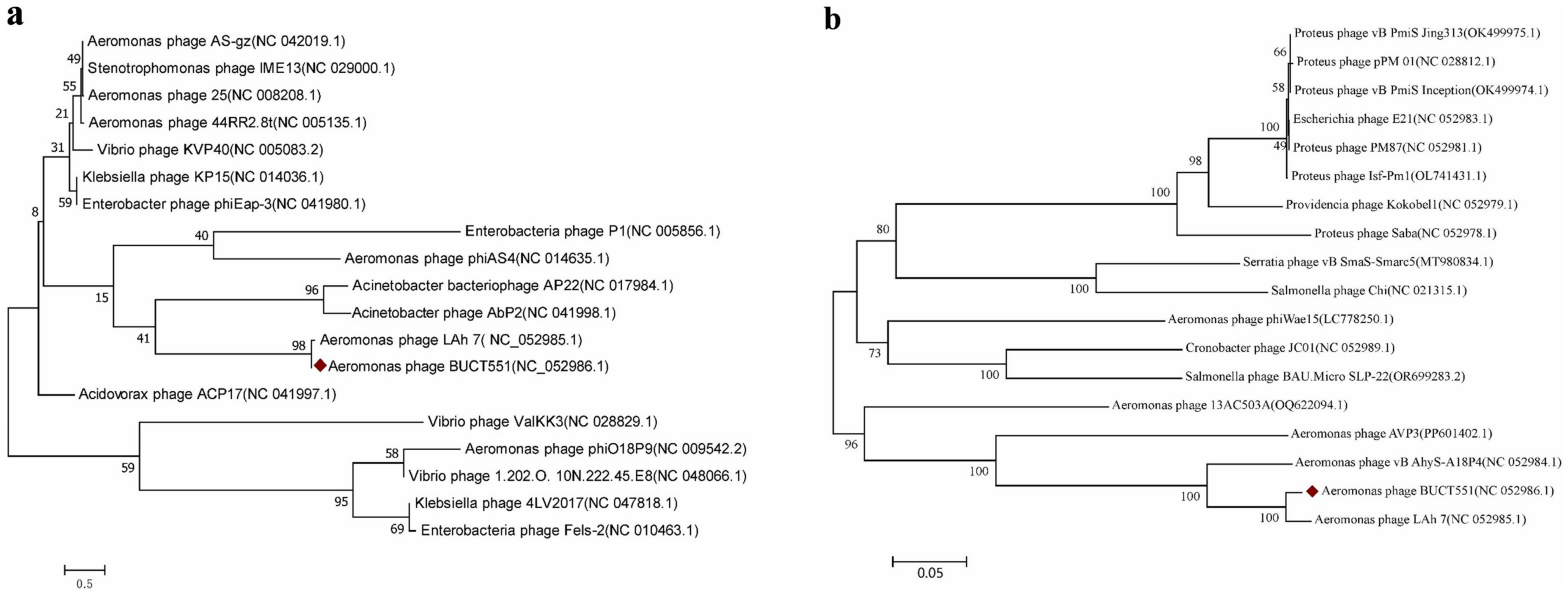
The phylogenetic tree was constructed using **(a)** capsid proteins and **(b)** terminase large subunit. Phylogenetic trees were constructed using the neighbor joining method of 1,000 bootstrap copies in MEGA7.

The major capsid and terminase large subunit proteins are commonly used as a marker gene to study phage evolutionary relationships (49–51). The evolutionary relationship between phage BUCT551 and other phages was determined based on the sequence of major capsid (ORF59) and terminase large subunit (ORF 64) to determine the phylogeny of BUCT551. Phylogenetic trees constructed based on the major capsid and large terminase subunit sequences both revealed that phage BUCT551 clustered within the same clade as phage LAh_7 (Figure 6), indicating a shared common ancestor. In accordance with the ICTV classification criteria, phage BUCT551 should be classified as a novel species within the genus *Sharonstreetvirus*.

### 3.9 *In vitro* phage efficacy in *Aeromonas* control

**Figure 7:**
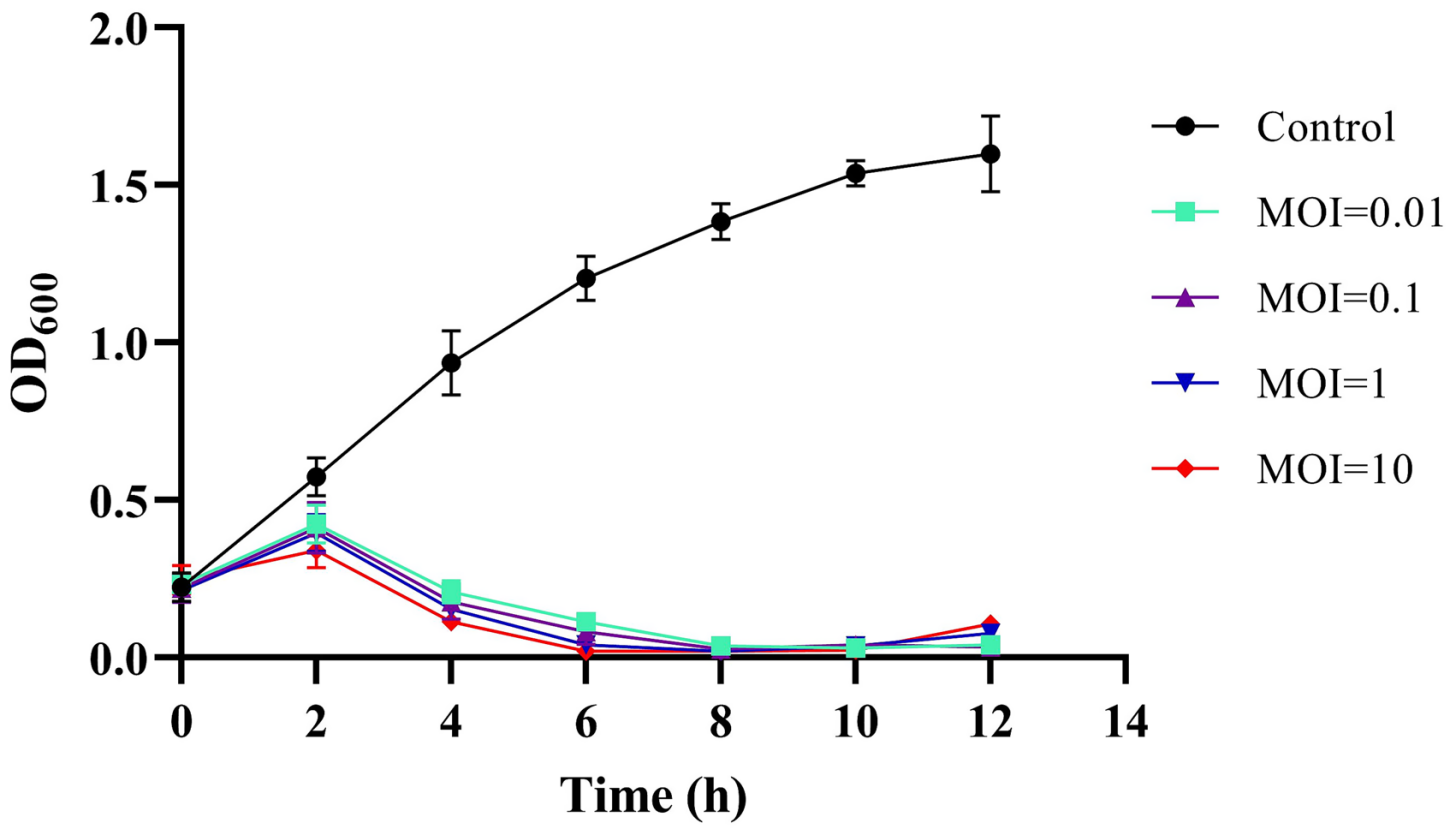
Bacteriolytic activity of phage BUCT551 at different MOIs against drug-resistance *A. hydrophila* strain Ah18 in liquid culture. Results are presented as mean values ± SD.

To further evaluate the application potential of phage BUCT551, its ability to inhibit the growth of *A. hydrophila* Ah18 was assessed *in vitro*. As shown in Figure 7, the absorbance of Ah18 cultured without phage continued to increase over 12 h. In contrast, the phage-treated groups exhibited an initial increase in OD₆₀₀ during the first 2 h, followed by a significant decline. By 6 h post-infection, OD₆₀₀ values in all MOI groups reached minimum levels. Moreover, from 6 to 12 h, the OD₆₀₀ remained stably minimized across all MOI conditions. These results demonstrate that phage BUCT551 effectively inhibits the growth of Ah18 *in vitro*, indicating its promising application potential.

## 4 Discussion

Advancements in aquaculture practices have promoted the intensification of cultivation systems, resulting in significantly elevated stocking densities of cultivated fish. However, this intensification process has concurrently increased the risk of disease outbreaks in cultivated fish populations (52). *A. hydrophila* is a well-established pathogen that infects a large range of cultured fish, including Atlantic salmon, trout, and turbot, with the main symptoms being tail bleeding and epidermis fester (5). At present, the main methods of prevention and treatment of *A. hydrophila* infection still depend on disinfectants and antibiotics. However, the long-term use of antibiotics may lead to drug resistance. Novel antibacterial therapies are therefore needed. As a novel type of antibacterial agent, phages offer advantages such as high specificity, no obvious side effects, easily to acquire, and leave no toxic residue (53). Phage therapy therefore provides a new alternative to control diseases caused by *A. hydrophila*.

In this study, a strain of *A. hydrophila* (designated Ah18) was isolated from an aquaculture facility culturing the yellow catfish (*Pelteobagrus fulvidraco*). Antimicrobial susceptibility testing revealed multidrug resistance in this strain (Table 1), which poses significant challenges for antibiotic-based therapy. To identify alternative therapeutic approaches, we conducted a systematic screening for lytic phages using strain Ah18 as the host bacterium. Phage BUCT551 against strain Ah18 was isolated through enrichment culture techniques followed by biological characterization, with the objective of developing phage-based interventions to combat antibiotic-resistant *A. hydrophila* s in aquaculture systems.

When considering tailored phage therapy, thorough characterization of individual phages is essential. TEM analysis of phage BUCT551 confirmed its morphological similarity to phages belonging to the *Siphoviridae* family, which is the most abundant taxonomic group within the *Caudoviricetes* class (54). Phage BUCT551 maintained high viability (≥95% titer retention) across 4–40 °C. Furthermore, pH stability tests indicated that phage activity remained relatively stable within a pH range of 5 to 10, aligning with the ecological tolerance of aquatic pathogens in the study environment. These biological characteristics indicated that phage BUCT551 has robust and stable bacteriolytic competence, which is an important basis for phage therapy. In the host range analysis assay, we revealed that five out of 16 strains of *Aeromonas* were susceptible to phage BUCT551. Interestingly, four out of these five strains were identified as *A. veronii*, which is also pathogenic to fish (55). Generally, phages exhibit a narrow host range, typically infecting only their indicator hosts, while a broad host range is often highly advantageous for combating polymicrobial infections (56). In previous studies, several *Aeromonas* phages also have been reported to exhibit a broad host spectrum. Wang et al. reported a lytic phage of *A. hydrophila* Aph2 that infected not only *A. hydrophila* but also *Aeromonas salmonicida* (57).

Typically, to ensure the suitability of phages as antimicrobial agents, comprehensive genotypic analysis is essential in addition to phenotypic characterization. Phages harboring antibiotic resistance genes (ARGs) or bacterial virulence genes (VGs) in their genomes may facilitate horizontal transfer of these genes during large-scale production or between bacterial hosts susceptible to phage infection. Consequently, the presence of ARGs or VGs is recognized as an unsuitable trait for phage-based preparations intended for therapeutic or biocontrol applications (58). Sequencing and analysis of phage genomes enhance the understanding of phage genomics and improve the reliability and safety of phages for therapeutic applications (59). Bioinformatic analysis of putative phage-encoded proteins revealed no known antibiotic resistance genes or virulence factor genes were found in phage BUCT551 genome. This absence of pathogenic elements, coupled with strictly lytic behavior observed in prior characterization, indicates that bacteriophage BUCT551 could potentially serve as a safe and targeted therapeutic agent against drug-resistant *A hydrophila* strain Ah18 infections.

Phage BUCT551 possesses a double-stranded DNA genome of 61,382 bp in length, with a GC content of 61.77%. In the phage BUCT551 genome, no genes associated with viral genome integration (such as genes encoding an integrase) were detected (Table 3), implying that it is a lytic phage. Through the identified predicted proteins, we found that phage BUCT551 encodes a N-6-adenine-methyltransferase (ORF35), which plays a vital role in evading bacterial defense systems (60, 61). Previous *in vitro* studies have demonstrated that phage-encoded methyltransferases can protect phage DNA from cleavage by host restriction endonucleases (41, 62). N-6-adenine-methyltransferase methylates the genome of phage BUCT551, which may facilitate evasion of the restriction–modification phage defense system of the host.

Through genomic sequencing and comparative genomic analysis, combined with phylogenetic trees constructed based on the highly conserved terminase large subunit and capsid protein genes, we demonstrate that phage BUCT551 belongs to the genus *Sharonstreetvirus* within the class *Caudoviricetes*. It is noteworthy that although the NCBI database contains extensive genomic data from *Aeromonas* phages, the genomic sequence similarity between BUCT551 and its closest relative (*Aeromonas* phage Lah_7) is only 86.75%, which is well below the 95% species demarcation threshold established by the ICTV (63)—supporting its classification as a novel species. Comprehensive characterization of BUCT551 thus advances our understanding of *Aeromonas* phages diversity, with implications for phage-based biocontrol strategies in aquaculture.

## 5 Conclusion

As a result of the large-scale use of antibiotics, the application of phage to control bacterial infections of fish in aquatic environments offers a sustainable and safer alternative. In this study, the *A. hydrophila* phage BUCT551 was identified and characterized. Our results indicated that phage BUCT551, along with other *A. hydrophila* phages, has great potential for use in phage cocktail therapy, or in combination with antibiotics and other disinfection strategies. Given the lack of identified *A. hydrophila* phages, exploring the large number of hypothetical proteins encoded within its genome will be the priority of our future research.

## Data Availability

All data generated for this study are included in the article/Supplementary material. NCBI accession number of BUCT551 nucleic acid sequence is NC_052986.1.

## Footnotes

^1^ https://rast.nmpdr.org/

^2^ https://www.ncbi.nlm.nih.gov/orffinder/

^3^ https://blast.ncbi.nlm.nih.gov/Blast.cgi

## References

[1] Kali A Kalaivani R Charles P Seetha KS. *Aeromonas hydrophila* meningitis and fulminant sepsis in preterm newborn: a case report and review of literature. Indian J Med Microbiol. (2016) 34:544–7. doi: 10.4103/0255-0857.195383 27934841

[2] Rasmussen-Ivey CR Figueras MJ Mcgarey D Liles MR. Virulence factors of *Aeromonas hydrophila*: in the wake of reclassification. Front Microbiol. (2016) 7:1337. doi: 10.3389/fmicb.2016.01337 27610107 PMC4997093

[3] Liu D Zhang T Wang Y Muhammad M Xue W Ju J. Knockout of alanine racemase gene attenuates the pathogenicity of *Aeromonas Hydrophila*. BMC Microbiol. (2019) 19:72. doi: 10.1186/S12866-019-1437-3, PMID: 30940083 PMC6444436

[4] Pu W Guo G Yang N Li Q Yin F Wang P. Three species of Aeromonas (*A. dhakensis*, *A. hydrophila* and *A. jandaei*) isolated from freshwater crocodiles (*Crocodylus siamensis*) with pneumonia and septicemia. Lett Appl Microbiol. (2019) 68:212–8. doi: 10.1111/Lam.13112, PMID: 30609084

[5] El-Bahar HM Ali NG Aboyadak IM Khalil S Ibrahim MS. Virulence genes contributing to *Aeromonas hydrophila* pathogenicity in *Oreochromis niloticus*. Int Microbiol. (2019) 22:479–90. doi: 10.1007/s10123-019-00075-3 30989358

[6] Igbinosa IH Igumbor EU Aghdasi F Tom M Okoh AI. Emerging *Aeromonas* species infections and their significance in public health. Sci World J. (2012) 2012:625023. doi: 10.1100/2012/625023 PMC3373137 22701365

[7] Citterio B Francesca B. *Aeromonas hydrophila* virulence. Virulence. (2015) 6:417–8. doi: 10.1080/21505594.2015.1058479 26055576 PMC4601520

[8] Stratev D Odeyemi OA. Antimicrobial resistance of *Aeromonas hydrophila* isolated from different food sources: a mini-review. J Infect Public Health. (2016) 9:535–44. doi: 10.1016/j.jiph.2015.10.006 26588876

[9] Tu VQ Nguyen TT Tran X Millard AD Phan HT Le NP. Complete genome sequence of a novel lytic phage infecting *Aeromonas hydrophila*, an infectious agent in striped catfish (*Pangasianodon hypophthalmus*). Arch Virol. (2020) 165:2973–7. doi: 10.1007/S00705-020-04793-2 32886215

[10] Yamada S Matsushita S Dejsirilert S Kudoh Y. Incidence and clinical symptoms of Aeromonas-associated travellers' diarrhoea in Tokyo. Epidemiol Infect. (1997) 119:121–6. doi: 10.1017/S0950268897007942, PMID: 9363009 PMC2808832

[11] Anjur N Sabran SF Daud HM Othman NZ. An update on the ornamental fish industry in Malaysia: *Aeromonas hydrophila*-associated disease and its treatment control. Vet World. (2021) 14:1143–52. doi: 10.14202/Vetworld.2021.1143-1152 34220115 PMC8243671

[12] Hasan O Khan W Jessar M Pathan AZ Lakdawala RH. Bone graft donor site infection with a rare organism, *Aeromonas hydrophila*. A typical location, presentation and organism with 2 years follow-up. Case report. Int J Surg Case Rep. (2018) 51:154–7. doi: 10.1016/J.Ijscr.2018.08.037 30172053 PMC6122150

[13] Manyi-Loh C Mamphweli S Meyer E Okoh A. Antibiotic use in agriculture and its consequential resistance in environmental sources: potential public health implications. Molecules. (2018) 23:795. doi: 10.3390/Molecules23040795, PMID: 29601469 PMC6017557

[14] Chen J Sun R Pan C Sun Y Mai B Li QX. Antibiotics and food safety in aquaculture. J Agric Food Chem. (2020) 68:11908–19. doi: 10.1021/Acs.Jafc.0c03996 32970417

[15] Elbehiry A Marzouk E Abdeen E Al-Dubaib M Alsayeqh A Ibrahem M. Proteomic characterization and discrimination of Aeromonas species recovered from meat and water samples with a spotlight on the antimicrobial resistance of *Aeromonas Hydrophila*. Microbiology. (2019) 8:E782. doi: 10.1002/Mbo3.782, PMID: 30614207 PMC6854848

[16] Uechi K Tada T Sawachi Y Hishinuma T Takaesu R Nakama M. A carbapenem-resistant clinical isolate of *Aeromonas Hydrophila* in Japan harbouring An acquired gene encoding Ges-24 β-lactamase. J Med Microbiol. (2018) 67:1535–7. doi: 10.1099/Jmm.0.000842, PMID: 30289383

[17] Pires DP Costa AR Pinto G Meneses L Azeredo J. Current challenges and future opportunities of phage therapy. FEMS Microbiol Rev. (2020) 44:684–700. doi: 10.1093/Femsre/Fuaa017 32472938

[18] Gordillo Altamirano FL Barr JJ. Phage therapy in the postantibiotic era. Clin Microbiol Rev. (2019) 32:E00066-18. doi: 10.1128/CMR.00066-18, PMID: 30651225 PMC6431132

[19] Lin DM Koskella B Lin HC. Phage therapy: an alternative to antibiotics in the age of multi-drug resistance. World J Gastrointest Pharmacol Ther. (2017) 8:162–73. doi: 10.4292/wjgpt.v8.i3.162, PMID: 28828194 PMC5547374

[20] Kortright KE Chan BK Koff JL Turner PE. Phage therapy: a renewed approach to combat antibiotic-resistant bacteria. Cell Host Microbe. (2019) 25:219–32. doi: 10.1016/J.Chom.2019.01.014 30763536

[21] Cooper CJ Khan Mirzaei M Nilsson AS. Adapting drug approval pathways for bacteriophage-based therapeutics. Front Microbiol. (2016) 7:1209. doi: 10.3389/fmicb.2016.01209, PMID: 27536293 PMC4971087

[22] Chan BK Abedon ST Loc-Carrillo C. Phage cocktails and the future of phage therapy. Future Microbiol. (2013) 8:769–83. doi: 10.2217/Fmb.13.47 23701332

[23] Anastasiou E Lorentz KO Stein GJ Mitchell PD. Prehistoric schistosomiasis parasite found in the Middle East. Lancet Infect Dis. (2014) 14:553–4. doi: 10.1016/S1473-3099(14)70794-7 24953264

[24] Zhao R Wang J Wang D Wang Y Hu G Li S. Isolation, identification, and characterisation of a novel St2378 *Aeromonas hydrophila* strain from naturally diseased frogs, *Rana dybowskii*. Pathogens. (2024) 13:552. doi: 10.3390/Pathogens13070552 39057779 PMC11279971

[25] Han P Hu Y An X Song L Fan H Tong Y. Biochemical and genomic characterization of a novel bacteriophage Buct555 lysing *Stenotrophomonas Maltophilia*. Virus Res. (2021) 301:198465. doi: 10.1016/J.Virusres.2021.198465, PMID: 34052250

[26] Chen Y Sun E Song J Yang L Wu B. Complete genome sequence of a novel T7-like bacteriophage from a *Pasteurella multocida* capsular type a isolate. Curr Microbiol. (2018) 75:574–9. doi: 10.1007/S00284-017-1419-3 29307051

[27] Han P Zhang W Pu M Li Y Song L An X. Characterization of the bacteriophage Buct603 and therapeutic potential evaluation against drug-resistant *Stenotrophomonas Maltophilia* in a mouse model. Front Microbiol. (2022) 13:906961. doi: 10.3389/Fmicb.2022.906961, PMID: 35865914 PMC9294509

[28] Wang R Cong Y Mi Z Fan H Shi T Liu H. Characterization and complete genome sequence analysis of phage Gp4, a novel lytic Bcep22-like Podovirus. Arch Virol. (2019) 164:2339–43. doi: 10.1007/S00705-019-04309-7, PMID: 31214785

[29] Holst Sørensen MC Van Alphen LB Fodor C Crowley SM Christensen BB Szymanski CM. Phase variable expression of capsular polysaccharide modifications allows *Campylobacter jejuni* to avoid bacteriophage infection in chickens. Front Cell Infect Microbiol. (2012) 2:11. doi: 10.3389/Fcimb.2012.00011 22919603 PMC3417653

[30] Yang Y Cai L Ma R Xu Y Tong Y Huang Y. A novel Roseosiphophage isolated from the oligotrophic South China Sea. Viruses. (2017) 9:109. doi: 10.3390/V9050109, PMID: 28505134 PMC5454422

[31] Margulies M Egholm M Altman WE Attiya S Bader JS Bemben LA. Genome sequencing in microfabricated high-density picolitre reactors. Nature. (2005) 437:376–80. doi: 10.1038/Nature03959 16056220 PMC1464427

[32] Bolger AM Lohse M Usadel B. Trimmomatic: a flexible trimmer for Illumina sequence data. Bioinformatics. (2014) 30:2114–20. doi: 10.1093/Bioinformatics/Btu170 24695404 PMC4103590

[33] Sullivan MJ Petty NK Beatson SA. Easyfig: a genome comparison visualizer. Bioinformatics. (2011) 27:1009–10. doi: 10.1093/Bioinformatics/Btr039 21278367 PMC3065679

[34] Lowe TM Chan PP. Trnascan-se on-line: integrating search and context for analysis of transfer rna genes. Nucleic Acids Res. (2016) 44:W54–7. doi: 10.1093/Nar/Gkw413 27174935 PMC4987944

[35] Liu B Zheng D Zhou S Chen L Yang J. Vfdb 2022: a general classification scheme for bacterial virulence factors. Nucleic Acids Res. (2022) 50:D912–912d917. doi: 10.1093/Nar/Gkab1107 34850947 PMC8728188

[36] Cosentino S Voldby Larsen M Møller Aarestrup F Lund O. Pathogenfinder--distinguishing friend from foe using bacterial whole genome sequence data. PLoS One. (2013) 8:E77302. doi: 10.1371/Journal.Pone.0077302 24204795 PMC3810466

[37] Kumar S Stecher G Tamura K. Mega7: molecular evolutionary genetics analysis version 7.0 for bigger datasets. Mol Biol Evol. (2016) 33:1870–4. doi: 10.1093/Molbev/Msw054 27004904 PMC8210823

[38] Liu J Gao S Dong Y Lu C Liu Y. Isolation and characterization of bacteriophages against virulent *Aeromonas Hydrophila*. BMC Microbiol. (2020) 20:141. doi: 10.1186/S12866-020-01811-W, PMID: 32487015 PMC7268745

[39] Tang C Deng C Zhang Y Xiao C Wang J Rao X. Characterization and genomic analyses of *Pseudomonas Aeruginosa* Podovirus Tc6: establishment of genus Pa11virus. Front Microbiol. (2018) 9:2561. doi: 10.3389/Fmicb.2018.02561, PMID: 30410478 PMC6209634

[40] Guilliam TA Keen BA Brissett NC Doherty AJ. Primase-polymerases are a functionally diverse superfamily of replication and repair enzymes. Nucleic Acids Res. (2015) 43:6651–64. doi: 10.1093/Nar/Gkv625 26109351 PMC4538821

[41] Kossykh VG Schlagman SL Hattman S. Phage T4 Dna [N6-adenine]methyltransferase. Overexpression, purification, and characterization. J Biol Chem. (1995) 270:14389–93. doi: 10.1074/Jbc.270.24.14389 7782299

[42] Prevelige PE Jr Cortines JR. Phage assembly and the special role of the portal protein. Curr Opin Virol. (2018) 31:66–73. doi: 10.1016/j.coviro.2018.09.004, PMID: 30274853

[43] Geagea H Labrie SJ Subirade M Moineau S. The tape measure protein is involved in the heat stability of *Lactococcus lactis* phages. Appl Environ Microbiol. (2018) 84:E02082-17. doi: 10.1128/Aem.02082-17 29150509 PMC5772233

[44] Pell LG Cumby N Clark TE Tuite A Battaile KP Edwards AM. A conserved spiral structure for highly diverged phage tail assembly chaperones. J Mol Biol. (2013) 425:2436–49. doi: 10.1016/J.Jmb.2013.03.035 23542344

[45] Dedeo CL Teschke CM Alexandrescu AT. Keeping it together: structures, functions, and applications of viral decoration proteins. Viruses. (2020) 12:1163. doi: 10.3390/V12101163 33066635 PMC7602432

[46] Jain D. Allosteric control of transcription in Gntr family of transcription regulators: a structural overview. IUBMB Life. (2015) 67:556–63. doi: 10.1002/Iub.1401, PMID: 26172911

[47] Liu GF Wang XX Su HZ Lu GT. Progress on the Gntr family transcription regulators in bacteria. Yi Chuan. (2021) 43:66–73. doi: 10.16288/J.Yczz.20-245 33509775

[48] Tsfasman IM Lapteva YS Krasovskaya LA Kudryakova IV Vasilyeva NV Granovsky IE. Gene expression of lytic endopeptidases Alpa and Alpb from *Lysobacter* sp. Xl1 in pseudomonads. J Mol Microbiol Biotechnol. (2015) 25:244–52. doi: 10.1159/000381266 26138026

[49] Abrescia NG Bamford DH Grimes JM Stuart DI. Structure unifies the viral universe. Annu Rev Biochem. (2012) 81:795–822. doi: 10.1146/annurev-biochem-060910-095130, PMID: 22482909

[50] Nasir A Caetano-Anollés G. A phylogenomic data-driven exploration of viral origins and evolution. Sci Adv. (2015) 1:E1500527. doi: 10.1126/Sciadv.1500527, PMID: 26601271 PMC4643759

[51] Liu H Geagea H Rousseau GM Labrie SJ Tremblay DM Liu X. Characterization of the *Escherichia coli* virulent myophage St32. Viruses. (2018) 10:616. doi: 10.3390/V10110616 30405057 PMC6266442

[52] Bosworth B Ott B Torrans L. Effects of stocking density on production traits of channel catfish×blue catfish hybrids. N Am J Aquac. (2015) 77:437–43. doi: 10.1080/15222055.2015.1024363

[53] Palma M Qi B. Advancing phage therapy: a comprehensive review of the safety, efficacy, and future prospects for the targeted treatment of bacterial infections. Infect Dis Rep. (2024) 16:1127–81. doi: 10.3390/Idr16060092, PMID: 39728014 PMC11675988

[54] Adriaenssens EM Edwards R Nash J Mahadevan P Seto D Ackermann HW. Integration of genomic and proteomic analyses in the classification of the Siphoviridae family. Virology. (2015) 477:144–54. doi: 10.1016/J.Virol.2014.10.016 25466308

[55] Li T Raza S Yang B Sun Y Wang G Sun W. *Aeromonas veronii* infection in commercial freshwater fish: a potential threat to public health. Animals (Basel). (2020) 10:608. doi: 10.3390/Ani10040608 32252334 PMC7222775

[56] Chung KM Liau XL Tang SS. Bacteriophages and their host range in multidrug-resistant bacterial disease treatment. Pharmaceuticals (Basel). (2023) 16:1467. doi: 10.3390/Ph16101467 37895938 PMC10610060

[57] Wang JB Yu MS Tseng TT Lin LC. Molecular characterization of Ahp2, a lytic bacteriophage of *Aeromonas hydrophila*. Viruses. (2021) 13:477. doi: 10.3390/V13030477 33799428 PMC8001559

[58] Abedon ST Thomas-Abedon C. Phage therapy pharmacology. Curr Pharm Biotechnol. (2010) 11:28–47. doi: 10.2174/138920110790725410 20214606

[59] Philipson CW Voegtly LJ Lueder MR Long KA Rice GK Frey KG. Characterizing phage genomes for therapeutic applications. Viruses. (2018) 10:188. doi: 10.3390/V10040188 29642590 PMC5923482

[60] Zhang M Gou Z Qu Y Su X. The indispensability of methyltransferase-like 3 in the immune system: from maintaining homeostasis to driving function. Front Immunol. (2024) 15:1456891. doi: 10.3389/Fimmu.2024.1456891, PMID: 39416774 PMC11479892

[61] Muszewska A Steczkiewicz K Ginalski K. Dirs and Ngaro retrotransposons in Fungi. PLoS One. (2013) 8:E76319. doi: 10.1371/Journal.Pone.0076319, PMID: 24086727 PMC3783388

[62] Murphy J Mahony J Ainsworth S Nauta A Van Sinderen D. Bacteriophage orphan DNA methyltransferases: insights from their bacterial origin, function, and occurrence. Appl Environ Microbiol. (2013) 79:7547–55. doi: 10.1128/Aem.02229-13 24123737 PMC3837797

[63] Adriaenssens EM Sullivan MB Knezevic P Van Zyl LJ Sarkar BL Dutilh BE. Taxonomy of prokaryotic viruses: 2018-2019 update from the ICTV bacterial and archaeal viruses subcommittee. Arch Virol. (2020) 165:1253–60. doi: 10.1007/S00705-020-04577-8 32162068 PMC13502909

