# Supplementary material for: Isolation and genomic analysis of phage BUCT551 against drug-resistant Aeromonas hydrophila
Source: Front Vet Sci. 2025 Sep 26;12:1679093. doi: 10.3389/fvets.2025.1679093 (PMC12512217; doi:10.3389/fvets.2025.1679093)
Supplement: Supplementary file 1 [file Data_Sheet_1.DOCX]

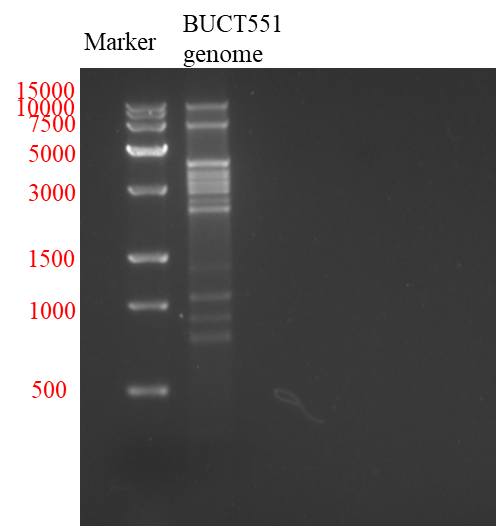


**Supplementary Figure S1.** BUCT551 whole genome digested by restriction enzyme EcoRⅠ, BamHⅠ and Hind Ⅲ.

**Supplementary Table 1** The lytic activity of phage BUCT551 against the strains related to the genus *Aeromonas*

| Strain | Genus, species | susceptibility |
| --- | --- | --- |
| 2195 | Vibrio, parahaemolyticus | not susceptible |
| 2196 | Vibrio, parahaemolyticus | not susceptible |
| 2197 | Vibrio, parahaemolyticus | not susceptible |
| 2198 | Vibrio, parahaemolyticus | not susceptible |
| 2199 | Vibrio, parahaemolyticus | not susceptible |
| 2200 | Vibrio, parahaemolyticus | not susceptible |
| 2201 | Vibrio, parahaemolyticus | not susceptible |
| 2202 | Vibrio, parahaemolyticus | not susceptible |
| 2203 | Vibrio, parahaemolyticus | not susceptible |
| 2204 | Vibrio, parahaemolyticus | not susceptible |
| 2273 | Vibrio, parahaemolyticus | not susceptible |
| 2274 | Vibrio, parahaemolyticus | not susceptible |
| 2275 | Vibrio, parahaemolyticus | not susceptible |
| 2276 | Vibrio, parahaemolyticus | not susceptible |
| 2277 | Vibrio, parahaemolyticus | not susceptible |
| 2210 | Vibrio, alginolyticus | not susceptible |
| 2211 | Vibrio, alginolyticus | not susceptible |
| 2212 | Vibrio, alginolyticus | not susceptible |
| 2213 | Vibrio, alginolyticus | not susceptible |
| 2214 | Vibrio, alginolyticus | not susceptible |
| 2215 | Vibrio, alginolyticus | not susceptible |
| 2216 | Vibrio, alginolyticus | not susceptible |
| 2217 | Vibrio, alginolyticus | not susceptible |
| 2218 | Vibrio, alginolyticus | not susceptible |
| 2219 | Vibrio, alginolyticus | not susceptible |
| 2220 | Vibrio, alginolyticus | not susceptible |
| 2221 | Vibrio, alginolyticus | not susceptible |
| 2222 | Vibrio, alginolyticus | not susceptible |
| 2223 | Vibrio, alginolyticus | not susceptible |
| 2224 | Vibrio, alginolyticus | not susceptible |
